# Distribution of control during bimanual movement and stabilization

**DOI:** 10.1038/s41598-024-67303-3

**Published:** 2024-07-17

**Authors:** Atsushi Takagi, Makio Kashino

**Affiliations:** NTT Communication Science Laboratories, 3-1 Morinosato Wakamiya, Atsugi, Kanagawa 243-0198 Japan

**Keywords:** Motor control, Neuroscience

## Abstract

In two-handed actions like baseball batting, the brain can allocate the control to each arm in an infinite number of ways. According to hemispheric specialization theory, the dominant hemisphere is adept at ballistic control, while the non-dominant hemisphere is specialized at postural stabilization, so the brain should divide the control between the arms according to their respective specialization. Here, we tested this prediction by examining how the brain shares the control between the dominant and non-dominant arms during bimanual reaching and postural stabilization. Participants reached with both hands, which were tied together by a stiff virtual spring, to a target surrounded by an unstable repulsive force field. If the brain exploits each hemisphere’s specialization, then the dominant arm should be responsible for acceleration early in the movement, and the non-dominant arm will be the prime actor at the end when holding steady against the force field. The power grasp force, which signifies the postural stability of each arm, peaked at movement termination but was equally large in both arms. Furthermore, the brain predominantly used the arm that could use the stronger flexor muscles to mainly accelerate the movement. These results point to the brain flexibly allocating the control to each arm according to the task goal without adhering to a strict specialization scheme.

## Introduction

Studies of one-handed or unimanual reaching movements of healthy and neurologically damaged patients suggest that the left and right arms may be controlled by different mechanisms^1–4^. This theory, known as hemispheric specialization^5,6^, proposes that the dominant hemisphere has a superior internal model of the arm^7^, which gives it an advantage in accurately directing movements during ballistic motion. The non-dominant hemisphere is specialized at postural stabilization, which could explain why the non-dominant limb is generally stiffer during movements^8,9^. However, a recent study showed that the size and direction of the dominant (D) and non-dominant (ND) arm’s reflex response was comparable, meaning that the internal models of the dominant and non-dominant arms are known adequately by the brain^10^. Another study looked at the adaptation rates of the dominant and non-dominant arms during unimanual reaching movements when experiencing a novel velocity-dependent force field, and found comparable learning rates and no significant difference in the stabilization or cocontraction of the arms during learning^11^. Thus, it remains unclear to what extent hemispheric specialization can explain the control of the dominant and non-dominant arms and under what conditions such interlimb differences might emerge.

Past studies have mainly examined the control of each arm separately, where the specialization of each hemisphere may be difficult to discern as both ballistic control and stabilization are needed to reach and stop at a target quickly. Physically coupled bimanual movements, on the other hand, give the brain a choice in how to divide the control between the arms. Imagine a scenario where a glass cup is held with both hands outside on a windy day. The cup can be moved by the dominant arm while the non-dominant arm relaxes, or vice versa, or both arms could contribute equally. The cup can be stabilized against random gusts of wind by stiffening just the dominant arm, just the non-dominant arm, or via a combination of the two. In such physically coupled bimanual tasks, the redundancy in the dynamics allows the brain to exploit each arm’s specialization so that the arm most suited to the task is used.

One study used a physically coupled bimanual reaching task to examine the dominant and non-dominant arm’s ability to stabilize against self-induced perturbations^12^, but because the roles of reaching and stabilization were pre-allocated beforehand by the experimenter, this study could not investigate how the brain divides the control between the arms. We have carried out two studies that examined how the brain shared the stabilization control between the arms when an object held with both wrists is destabilized by force perturbations, but these studies only examined the sharing of stabilization control^13,14^.

**Figure 1:**
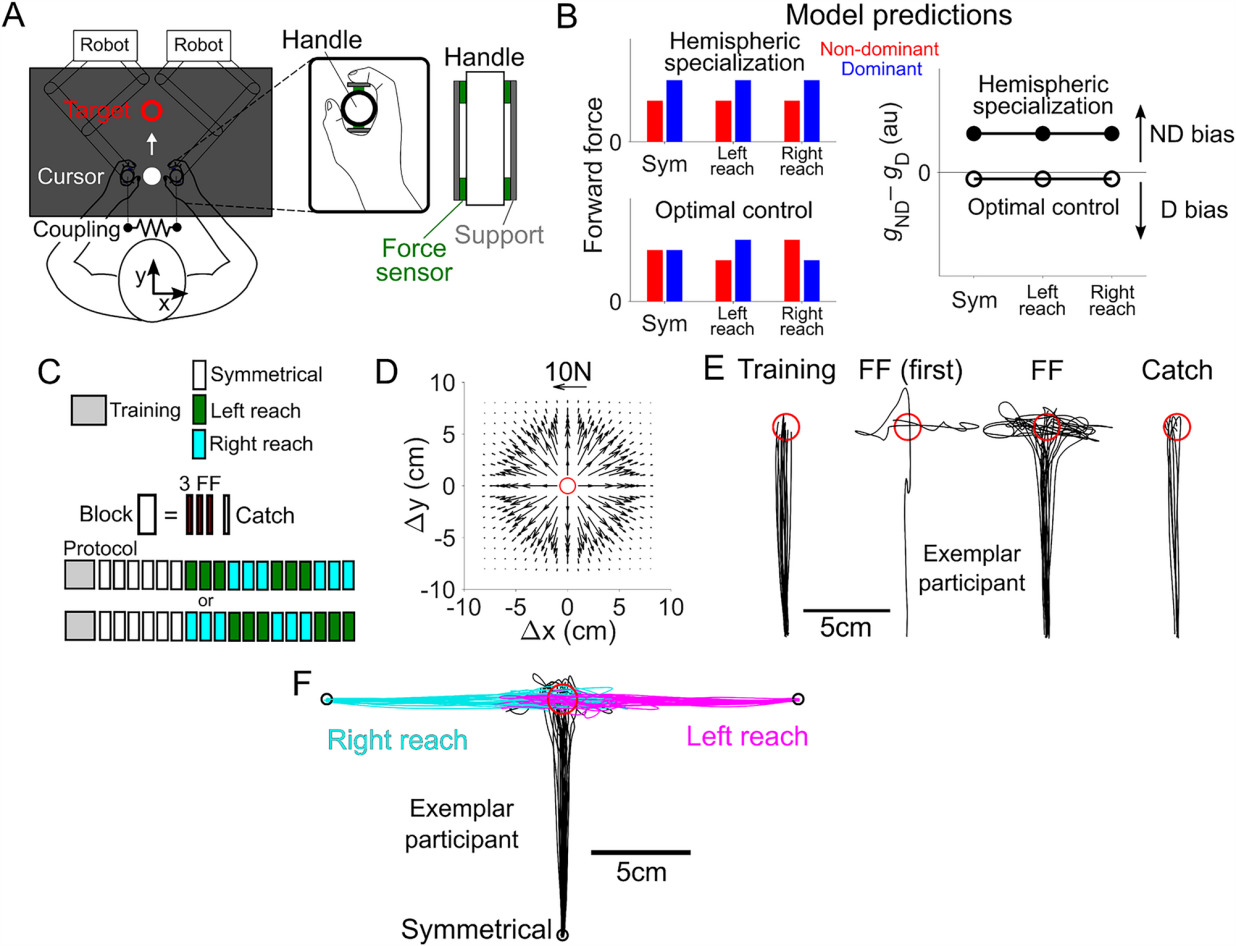
Coupled bimanual movement and stabilization to test hemispheric specialization. (**A**) Schematic of the experimental setup. Hands were physically coupled by a strong virtual spring. Each hand’s power grasp force was recorded by four pancake force sensors. (**B**) Hemispheric specialization and optimal control’s predictions of how the forward force and the grasp force should be distributed between the arms. (**C**) Experimental protocol consisted of training then symmetrical blocks followed by either left or right reaching blocks whose ordering was counterbalanced (n = 6 followed upper protocol, n = 6 lower). (**D**) Unstable force field located at the center of the target was imposed on force field trials demanding postural stabilization. In catch trials, the force field was abruptly removed. (**E**) Cursor trajectories from an exemplar participant from symmetrical reaches towards a target (red circle). Training, the first force field trial, subsequent FF trials, and catch trials are shown. (**F**) Cursor trajectories from the same exemplar participant during symmetrical (black), left (magenta) and right reaching (teal).

Here, we designed a reaching and stabilization task where the brain could divide the control between the arms in any number of ways. The two hands were connected by a stiff virtual spring, and participants had to reach and stop at a target location. On some trials, an unstable force field was placed at the target location, which pushed the arms away from the target. To successfully complete the task, participants had to reach, stabilize, and stop at the target location. This task can be broken down into a reach and stabilization phase. The chief actor during the ballistic reaching phase was quantified by the dominant and non-dominant arm’s forward force, defined as the force in the direction of the movement, during the initial phase of reaching. The chief stabilizer during the movement was measured by the size of the dominant and non-dominant arm’s power grasp force (Fig. 1A). Recently, we showed that the arm’s power grasp force is modulated in response to environmental perturbations^15^ and stays constant during reaching movements. This is unlike the pinch grip force, or the force between the thumb and index fingers, which increases in response to tangential forces^16^. Furthermore, an increase in the power grasp linearly increases the arm’s endpoint stiffness^17^, making it an ideal method suited to measuring the arm’s stability.

Hemispheric specialization expects the dominant arm to mainly contribute towards ballistic motion due to its specialization in controlling rapid goal-directed movements, while the non-dominant arm should be mainly responsible for holding a stable posture as the non-dominant side is specialized at stabilization. It predicts the forward force in the dominant arm to be larger than the non-dominant arm during the ballistic reaching phase, and it also predicts the non-dominant arm’s grasp force to be larger when stabilizing and stopping at the target (Fig. 1B, top left).

Optimal control theory has also been proposed to explain how the brain shares the control of the two arms during bimanual movements^18^. In optimal control, the two arms are controlled to minimize error and total effort^19^. One study examined how the left and right fingers were used together to produce an isometric force, which found that the contributions of the left and right arms depended on the strength of the acting limbs, thereby minimizing the total effort across both limbs^20^. Since the arm’s flexor muscles are known to be 50–100% stronger than the extensor muscles^21^, optimal control theory predicts that the brain will mainly utilize the arm that can use the stronger flexor muscles in the ballistic reaching phase. To test this prediction, we incorporated symmetric, left, and right directed reaching movements in our task. The dominant arm’s stronger flexor muscle can be used in left reaching and vice versa for right reaching, so optimal control predicts greater forward force in the dominant arm during left reaching and vice versa (Fig. 1B, bottom left). Hemispheric specialization predicts greater forward force in the dominant arm for all movement directions because the dominant hemisphere is specialized towards ballistic control.

Since the dominant arm’s maximum power grasp is roughly 10% stronger than the left arm’s^22,23^, optimal control expects a somewhat greater contribution of the dominant arm during stabilization (Fig. 1B, right). In contrast, hemispheric specialization expects the non-dominant arm’s power grasp to be larger because it is better suited at stabilization (Fig. 1B, right).

## Material and methods

### Experiment protocol

The experiment was approved by the institutional ethics committee (ID: R05-014), was performed in accordance with the relevant guidelines at NTT Communication Science Laboratories in Japan with all participants providing written informed consent prior to participation.

12 right-handed participants (36.8 ± 1 years, all male) with no known motor impairments partook in the experiment. The Edinburgh Handedness Inventory was used to confirm the right-handedness of our participants (0.96 ± 0.02).

Participants grabbed onto the two handles of the bimanual robotic interface (KINARM, BKIN Technologies) and sat with their midline aligned with the center of the task workspace where the target would be displayed. Since the two handles moved within a plane at the same vertical height, the hand positions were displaced along the horizontal *x* axis by $d/2=15$ cm, which was fixed throughout the experiment. The robotic interfaces were programmed to couple the arms rigidly via a spring-like force. The right handle experienced a force

1 $${\mathbf{F}}_{R}=1000\left(\genfrac{}{}{0pt}{}{\left({x}_{L}-d\right)-\left({x}_{R}+d\right)}{{y}_{L}-{y}_{R}}\right)+10\left(\genfrac{}{}{0pt}{}{{\dot{x}}_{L}-{\dot{x}}_{R}}{{\dot{y}}_{L}-{\dot{y}}_{R}}\right),$$

where $\left({x}_{L},{y}_{L}\right)$ and $\left({x}_{R},{y}_{R}\right)$ are the positions of the left and right handles, respectively. The force on the left arm was ${\mathbf{F}}_{L}=-{\mathbf{F}}_{R}$. This force was active throughout the experiment.

Four three-axis force sensors (USL-H5-200N, Tec Gihan) were used to measure each arm’s power grasp force (eight total sensors for both arms). Two sensors were connected by a 3D printed bar with dimensions of 10 × 0.1x0.3 cm through which the participant applied the force. Two such bars were placed on either side of the handle (Fig. 1A). Participants grasped the handle by placing the distal phalanges on one bar and the center of the palm on the other bar. The readings from the distal phalange force sensors ${g}_{DP1}$ and ${g}_{DP2}$ and those from the palm ${g}_{P1}$ and ${g}_{P2}$ were summed together to obtain an estimate of the grasp force

2 $$g=\text{min}\left({g}_{DP1}+{g}_{DP2},{g}_{P1}+{g}_{P2}\right)\text{N}\,.$$

The grasp force for the non-dominant left arm ${g}_{\text{ND}}$ and the dominant arm ${g}_{\text{D}}$ were calculated separately according to the above equation.

An unstable force field was centered at the target position $\left({x}_{t},{y}_{t}\right)$ that perturbed the mean position of the hands $x=0.5({x}_{L}+{x}_{R})$ and $y=0.5({y}_{L}+{y}_{R})$ (Fig. 1D). The perturbing force was

3 $${\mathbf{F}}_{FF}=750 {e}^{-{(x}^{2}{+y}^{2})/{c}^{2}}\left(\genfrac{}{}{0pt}{}{x}{y}\right)\text{ N}$$

where $c=0.04$. ${\mathbf{F}}_{FF}$ was exerted on each arm during force field trials so each arm experienced the same perturbing force. In other words, the force field’s direction and size was determined by the average position of the two hands, and the resultant force field was applied on both arms. An increase in the grasp force in either arm could simply be a reaction to the force from the unstable force field, instead of a genuine response from the brain dividing up the stabilizing control between the arms. To remedy this problem, every fourth trial was a catch trial where the unstable force field was removed abruptly by setting ${\mathbf{F}}_{FF}=0$. If the grasp force in force field trials and catch trials is similar, then the division of stabilization control between the arms is likely to be voluntarily controlled by the brain in a feedforward manner.

The final target position was the same for all three movement directions, which was located at ${x}_{t}=0$ and ${y}_{t}=15$ cm. For symmetrical movements, the starting position was $(\text{0,3})$ cm, for left reaching it was $(\text{12,15})$ cm, and for right reaching it was set to $(-\text{12,15})$ cm. The distance from the starting position to the target was 12 cm for all movement directions. The duration of each movement was provided as feedback to participants on all trials. When the movement duration was shorter than 400 ms, a ‘fast’ message was displayed on-screen, while for movements longer than 600 ms a ‘slow’ message was shown. For movements between 400 and 600 ms, no message was displayed. The trial ended when the mean position of the hands was inside the target circle and had a speed slower than 3 cm/s.

Participants were split into two groups. Each group experienced a different ordering of trials (Fig. 1C). All participants first completed 15 training trials where ${\mathbf{F}}_{FF}=0$. Then they completed six symmetrical blocks, where each block was composed of three consecutive force field trials followed by a catch trial. Half of our participants then experienced left reaching (LR) and right reaching (RR) blocks in the order {3 LR, 3 RR, 3 LR, 3 RR}. The other half completed them in the order {3 RR, 3 LR, 3 RR, 3 LR} to counterbalance the effects of learning across movements. The entire experiment was composed of 87 trials.

### Statistical analysis

**Figure 2:**
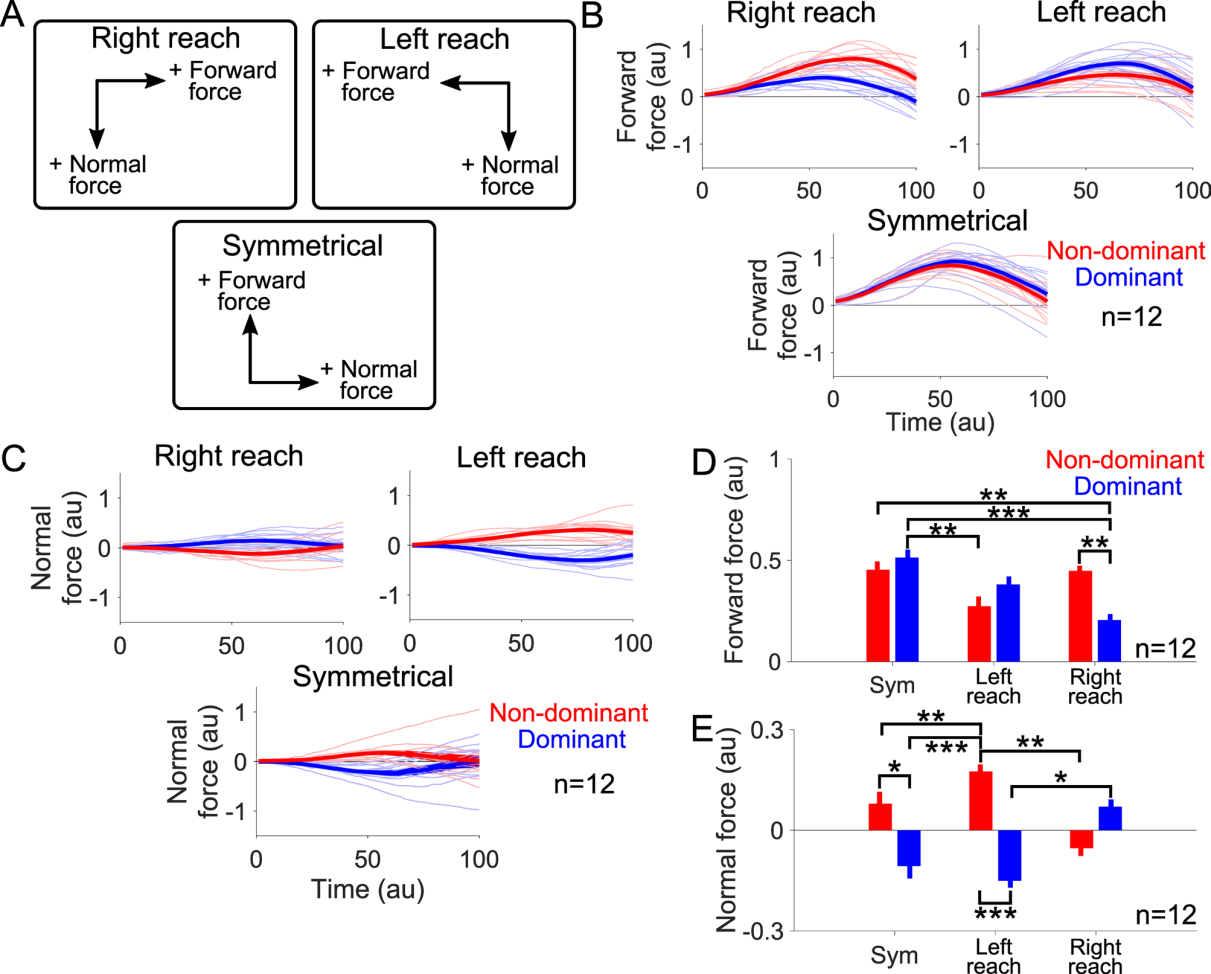
Each arm’s contribution towards forward and normal forces during the initial phase of reaching depended on movement direction. (**A**) Positive forward and normal force directions for each movement direction. (**B**) Forward force time-series (non-dominant arm in red, dominant arm in blue) in the three movement directions from all participants (mean ± SEM). Data comes from catch trials only and is time normalized from movement start to moment of peak velocity. (**C**) Normal force time-series, which was time normalized from movement start to time of peak velocity, from all participants also depended on movement direction. (**D**) Mean forward force from catch trials as a function of the movement type. The non-dominant arm produced significantly more forward force than the dominant arm during right reaching while the opposite trend was observed in both symmetrical and left reaching movements. * signifies p < 0.05, ** signifies p < 0.01, and *** denotes p < 0.001. (E) Mean normal force also depended on movement direction.

The force was analyzed separately along two different directions only during the ballistic phase, which was between the time of movement onset and time of peak velocity. The forward force is defined as the mean force along the direction of movement from movement start to the time of peak velocity (Fig. 2A). For symmetrical movements the forward force was along the + y axis, and during left and right reaching movements it was along the −x and + x axes, respectively. The normal force is defined as the mean force perpendicular to the movement direction from the start of the movement until the moment of peak velocity. In symmetrical movements it was oriented along the x axis, and in left and right reaching it was along the y axis (Fig. 2A). The forward and normal forces from only the catch trials were included in the analysis to exclude the forces that may have come from the force field.

The grasp force was analyzed in three different phases. The baseline phase was before the target was presented. The ballistic phase was between the time of movement onset and time of peak velocity. The holding phase was at the end of the movement when the cursor was inside the target.

The forward, normal, and grasp force were scaled for between-participant comparisons as Anderson–Darling tests revealed that they violated normality without rescaling. To rescale the variables, the mean value of each variable was calculated per force field trial, then each variable was divided by the average peak force magnitude $\overline{F }$ from movement start to time of peak velocity from all force field and catch trials. This process was repeated for each participant separately. For example, the forward force $F(i)$ from the *i*th trial was scaled to

4 $${F}^{\left(\text{N}\right)}(i)=F(i)/\overline{F } .$$

Anderson–Darling tests showed that all scaled variables were normally distributed.

Two-way repeated measures ANOVAs were performed with the arm (dominant arm, non-dominant arm) and the movement direction (symmetrical, left, right) as categorical factors to determine their effects on the forward force, normal force, and the grasp force. Differences between groups were tested using Tukey’s method to control for multiple comparisons.

## Results

The cursor’s trajectory or the movement of the mean position of the two hands extended straight towards the target during the training trials (Fig. 1E). On the first force field trial imposed during symmetrical movements, the exemplar participant’s cursor was pushed back to their surprise. Their cursor overshot the target position several times before coming to a stop at the target. On subsequent force field trials, the cursor position tended to deviate along the horizontal *x* axis due to the unstable force field. The cursor trajectory went straight to the target without deviation during catch trials when the unstable force field was abruptly removed. During left and right reaching, the cursor once again tended to deviate along the *x* axis despite the force field pushing the cursor with equal force along the *x* and *y* axes. This difference comes from the configuration of the arm’s endpoint stiffness, which is large along the *y* axis as the hand is moved in front of the body’s midline^17,24,25^. The cursor trajectories reveal that the unstable force field was successful at perturbing the end of the movement and demanded postural stabilization to overcome it to reach the target.

First, we examined how the dominant and non-dominant arms contributed to the reaching force during the ballistic phase of the task. We defined the ballistic phase as the time from movement initiation until the moment of peak velocity. The force was analyzed separately along two different directions. The forward force is defined as the force along the direction of movement (Fig. 2A). For symmetrical movements the forward force was along the + *y* axis, and during left and right reaching movements it was along the −*x* and + *x* axes, respectively. The normal force is defined as the force perpendicular to the movement direction. In symmetrical movements it was oriented along the + *x* axis, and in left and right reaching it was along the −*y* axis (Fig. 2A).

We examined the forward force of the non-dominant (red) and dominant (blue) arms during symmetrical, left and right reaching separately from movement beginning to the moment of peak velocity (Fig. 2B, see Figure S1A in Supplementary Materials for time-series of the entire movement without time normalization). In symmetrical movements, the non-dominant and dominant arms contributed equally to the forward force. However, the contributions were unequal during left and right reaching. When reaching towards the target to the right, most of the forward force came from the non-dominant arm. And when reaching to the left, the dominant arm mainly contributed to the forward force. To quantify this difference, we performed a two-way repeated measures ANOVA to compare the effect of the arm (non-dominant, dominant) and movement direction (symmetrical, left reach, right reach) categorical factors on the forward force (Fig. 2D). This revealed a significant effect of the movement direction (F(2,22) = 47.3, p < 0.001) and the interaction between the arm and the movement direction factors on the forward force (F(2,22) = 9.9, p < 0.001). In right reaching, the non-dominant arm’s forward force was significantly greater than the dominant arm’s (Tukey’s HSD, p = 0.007). The opposite was observed during left reaching, but the difference in the forward force between the arms was not significant (p = 0.50). And in symmetric reaching, the dominant and non-dominant arms produced roughly equal amounts of forward force (p = 0.92).

Next, we examined the normal force as a function of normalized time from movement beginning to moment of peak velocity (Fig. 2C, see Figure S1B for raw time-series). In symmetrical movements, participants displayed a preference in flexing with both arms as indicated by the non-dominant arm’s positive normal force and the dominant arm’s negative normal force. As with the forward force, the normal force was also different between left and right reaching. In right reaching, the non-dominant arm pushed away from the body while the dominant arm pulled into it. The opposite trend was seen during left reaching where the dominant arm pushed away from the body. A two-way repeated measures ANOVA was performed with the arm and the movement direction as categorical predictors on the normal force (Fig. 2E). The arm (F(1,11) = 23.7, p < 0.001), the movement direction (F(2,22) = 34.0, p < 0.001), and their interaction all had a significant effect on the normal force (F(2,22) = 15.8, p < 0.001). Both arms pushed significantly towards the body’s midline during symmetrical reaching (comparison of non-dominant and dominant normal force in symmetric reaching, p < 0.04). During left reaching, the non-dominant arm pulled towards the body while the dominant arm pushed away with a significant difference in normal force (p < 0.001). In right reaching, the non-dominant arm pushed away from the body and the dominant arm pulled towards it, but the normal forces weren’t significantly different between the arms (p = 0.29).

**Figure 3:**
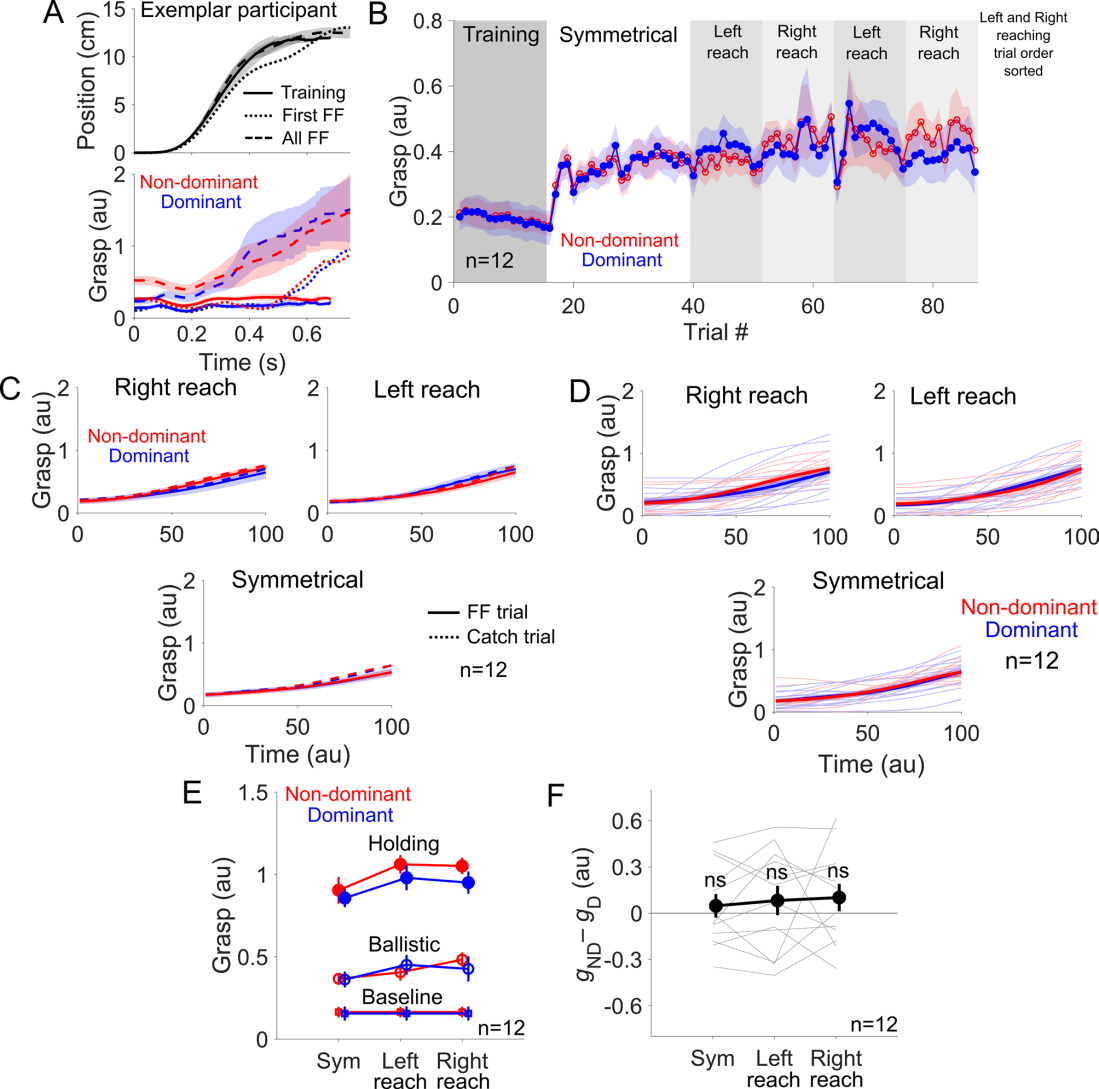
Participants had a within-subject preference in stabilizing with one arm. (**A**) Position and grasp force time-series (non-dominant arm in red, dominant arm in blue) from an exemplar participant (mean ± standard deviation) from all training trials, the first force field trial, then all subsequent force field trials from symmetrical movements. Grasp was initially low throughout the movement during training, but increased as stabilization was demanded. (**B**) Mean grasp force during entire motion phase as a function of trials, which jumped after force field trials were experienced. (**C**) Grasp force from movement start to moment of peak velocity plotted separately for all movement directions (mean ± SEM). Grasp force from catch (dashed lines) and force field trials (solid) were similar, indicating feedforward control of grasp. (**D**) Group mean (thick) and individual (thin) grasp force patterns from movement start to time of peak velocity in all movement directions. (**E**) Mean grasp force in all directions during baseline, ballistic, and holding phases of the movement. (**F**) Difference in mean grasp force in all movement directions from the holding phase. Directional biases were consistent within but not across participants. Population showed no bias in the grasp force.

We then looked at the grasp force during the physically coupled bimanual movements. The grasp force from an exemplar representative provides a glimpse into how the grasp force evolved as a function of time and over the course of the experiment (Fig. 3A). In training trials, the grasp force remained low and unchanged as the arms experienced no unpredictable forces. In the first force field trial, the grasp steadily increased after the force field resisted the motion. In subsequent force field trials, the participant had adapted to the force field by increasing their grasp force early in the movement. This increase in the grasp force is not a reaction to the force field because the time-normalized grasp force showed a similar steady increase during the movement even during catch trials where the force field was removed (Fig. 3C, dashed versus solid lines). Just like in a previous study of ours^15^, the change in the grasp force indicates an increase in the arm’s endpoint stiffness magnitude to stabilize the cursor at the end of the movement^17^. The mean grasp force during the entire duration of the movement is charted as a function of trials in Fig. 3B. Here, it can also be seen that the grasp force increased in the trials following the first force field trial 16, where it remained high throughout the task due to the unstable force field at the target.

The grasp force increased as a function of time in all movement directions (Fig. 3D). We calculated the mean grasp force in three phases. The baseline phase was before the target was presented. The ballistic phase was between the time of movement onset and time of peak velocity. Finally, the holding phase was at the end of the movement when the cursor was inside the target (see Figure S1C in the Supplementary Materials). A clear increase in grasp force was observed across these phases, but the non-dominant and dominant arm’s grasp force increased in an equal fashion (Fig. 3E). We performed a two-way repeated-measures ANOVA to compare the effect of the arm (non-dominant, dominant) and the movement direction (symmetrical, left reach, right reach) on the grasp force during the holding phase where the most difference was detected, but none of the factors significantly influenced it. A plot of the difference between the non-dominant minus the dominant arm’s grasp force reveals why (Fig. 3F). Our right-handed participants displayed a mixed preference in stabilizing with either arm. Some participants displayed a preference in grasping stronger with the non-dominant arm while others did so with their dominant arm. Therefore, as a group, no consistent effect could be found. However, most participants who displayed either a strong non-dominant or dominant arm grasp preference did maintain it in all movement directions.

## Discussion

We devised a task that demanded both movement and stabilization with both arms to examine how the brain shared the control between each arm during select phases of the movement. Our main findings were that the force exerted by each arm depended on the movement direction, and the grasp force increased in both hands equally to stabilize posture against the unstable force field.

The distribution of the forward force amongst the non-dominant and dominant arms, which depended on the movement direction, demonstrates the brain’s flexibility at using the muscles most appropriate to the task demands during bimanual movements. Since the flexor muscles are stronger than the extensors^21^, total effort expenditure can be minimized by primarily using the stronger muscles to accelerate the movement. Therefore, the brain used primarily the non-dominant arm for right bimanual reaching and the dominant arm for left reaching. And in symmetrical movements, the brain efficiently used both arms equally for acceleration. These results are consistent with the predictions of optimal control theory^19^ and how the brain uses the appropriate muscle or arm according to the demands of the task. Our results are consistent with a previous study that found that when participants produced a force with the index and little fingers of both hands, a majority of the force was generated by the stronger finger regardless of handedness^20^.

While the non-dominant arm did provide greater forward force during right reaching as predicted by optimal control, the difference between the non-dominant and dominant arm’s forward force during left reaching was not significantly different. Furthermore, a large difference in the normal force was observed between the arms during left reaching but not with right reaching. These differences could be explained by a difference in the non-dominant and dominant arm’s ability to coordinate the movement with the other arm. During right reaching, the non-dominant arm is the chief actor providing the greatest positive forward force, meaning that the dominant arm plays a subversive role where it must follow the non-dominant arm’s lead. This does not mean that the dominant arm can be passive. Instead, the dominant arm must deftly follow and keep up with the non-dominant arm’s position. The dominant arm is successful at following the non-dominant arm, which is why the forward force time-series of both arms are synchronized in time (Figure S1A in Supplementary Materials) and the normal force is minimal (Figure S1B). However, in left reaching, where the dominant arm is the prime actor and the non-dominant arm must follow the dominant arm’s lead, the non-dominant arm does not keep up with the dominant arm’s movement, which is why the non-dominant arm’s forward force is not as synchronized with the dominant arm’s forward force. Furthermore, a large normal force was observed, signifying some disagreement in the non-dominant and dominant arm’s positions during left reaching. However, it remains unclear why the ability to coordinate movements with the other arm depends on which arm is the prime actor. Further studies are needed to elucidate the difference in the non-dominant and dominant arm’s ability to follow the other arm during bimanual movements.

The grasp force increased significantly to stabilize the limbs in the presence of the unstable force field. As in a previous study of ours, this increase in the grasp force was not a reaction to the external forces, but was a deliberate control strategy to increase the limb’s impedance for stabilization^15^. While between-subject differences were observed, our population of right-handed participants increased the grasp force in both hands equally, a result that cannot be explained by either hemispheric specialization or by optimal control. Optimal control predicts a slight advantage of the dominant arm in stabilizing posture as it is roughly 10% stronger than the non-dominant arm in right-handed individuals^22,23^. However, the peak grasp force measured in our task was 33.2 ± 7.4 N (mean ± standard error). Since the mean maximum grasp force from right-handed male participants is approximately 842.8 N^22^, the peak grasp measured in our task constituted only 4% of the maximum grasp force, so a 10% difference in the maximum strength of the hands may not have affected our task. Further studies are needed to elucidate the mechanism that drives this difference in distribution of grasp control between the arms that is subject-specific.

While there are several reports of larger cocontraction, or a greater reliance on impedance control, in the non-dominant hand during finger tapping^8^ and elbow reaching^9^, it remains unclear if this is indicative of superior postural control or a way to compensate for the non-dominant limb’s poorer precision during rapid movements^26–28^. The non-dominant arm is less precise at moving towards the target direction^29^, and so it may have to correct for such errors by cocontracting or stiffening the limb towards the end of the movement.

Our study is the first to examine the distribution of control between the arms when moving and stabilizing the limbs, but several other related studies have examined the sharing of postural control between the arms. One study examined physically coupled bimanual movements to test which arm is better at holding a fixed posture while the other arm reached to a target when the hands were coupled together^12^. While the non-dominant arm’s position deviated less when holding a fixed posture, it was unclear whether this was due to superior stabilization or due to better prediction of the dominant arm’s force, thereby enabling the non-dominant arm to cancel-out the dominant arm’s force with greater precision. We have also conducted two earlier studies that examined how participants used their wrists to hold and stabilize a virtual object that oscillated violently at a high frequency^13,14^. One study found that the dominant wrist of left- and right-handed participants cocontracted more than the non-dominant one to stabilize the object^13^. The other study found that right-handed people displayed no strong preference in cocontracting with either wrist^14^, but across different tasks, participants maintained a consistent bias in the cocontraction between the wrists, so participants preferring to cocontract their non-dominant wrist did so across different conditions. Together with our results, this suggests that when the brain can freely share the control of stabilization between the arms, it does so in a roughly equal manner or by using the dominant arm slightly more. In essence, the brain prioritizes effort and strength when it must increase the total impedance of both arms.

A limitation of our study is in how the arm’s endpoint stiffness was estimated by measuring the hand’s power grasp force. While the grasp force is linearly related to the arm’s endpoint stiffness magnitude at different postures^17^, we cannot deny the possibility that participants could have changed their arm’s stiffness independently of the grasp force. However, we believe that such a strategy is inefficient because the arm’s stiffness cannot be transmitted to the robotic handle without increasing the grasp force first. Furthermore, the grasp force did increase substantially as the hands approached the center of the unstable force field, implying that our grasp force measure provides a sufficient estimate of the arm’s desire to increase stability.

Another limitation is the generalizability of our findings. Hemispheric specialization may hold for more complex movements three-dimensional movements like swinging a baseball bat or in tasks where one arm must complete a different action relative to the other, like when opening a jar. However, the unstable force field we used is not as artificial as it may seem since the same dynamics are experienced when pushing against a convex three-dimensional surface^30^. Furthermore, we deliberately set up a task without pre-assigned roles to gauge how the brain assigns the control to each arm in a redundant system, something that cannot be done with a task like opening a jar where pre-assigned roles are a given.

Finally, one thing that remains unclear is whether the control of stiffness or limb stability is different between the arms. If the difference in impedance control between the arms is smaller than the effort needed to maintain a difference in left–right arm stiffness, then we would not have observed a difference in grasp force between the arms. Further studies are needed to ascertain whether the non-dominant or dominant arm is better at modulating the level of cocontraction or stiffness.

### Supplementary Information

Supplementary Figure S1.

## Data Availability

The data is available on the Figshare repository (10.6084/m9.figshare.24970590).
