# Supplementary material for: Distribution of control during bimanual movement and stabilization
Source: Sci Rep. 2024 Jul 17;14:16506. doi: 10.1038/s41598-024-67303-3 (PMC11255328; doi:10.1038/s41598-024-67303-3)
Supplement: Supplementary file 1 — Supplementary Figure S1. [file 41598_2024_67303_MOESM1_ESM.docx]

**Supplementary Materials to “Distribution of control during bimanual movement and stabilization”**

A. Takagi^1^* and M. Kashino^1^

^1^NTT Communication Science Laboratories, 3-1 Morinosato Wakamiya, Atsugi, Kanagawa, 243-0198, Japan.

*corresponding author: atsushi.takagi@ntt.com

**
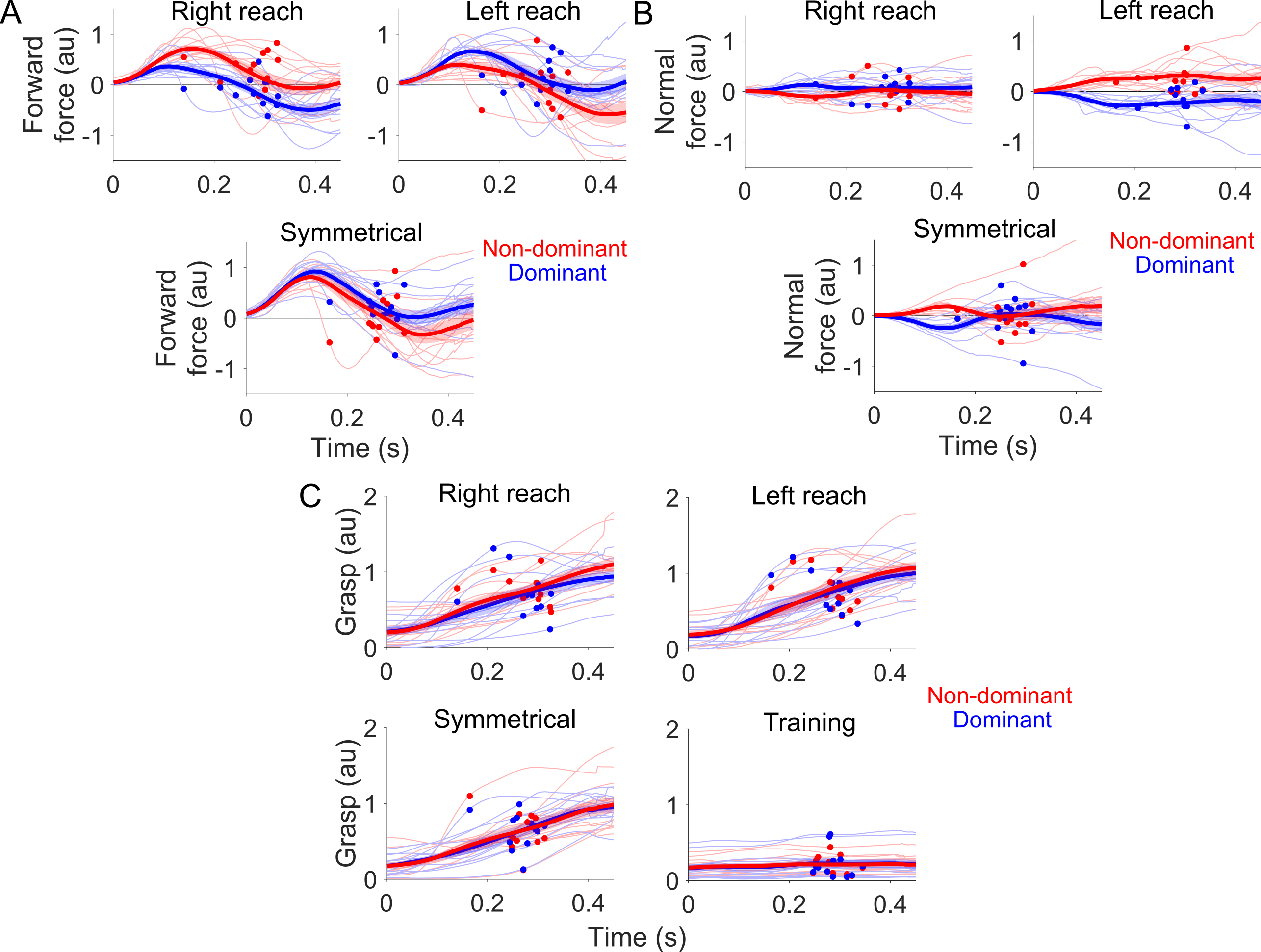
**

**Figure S1.** Forward, normal, and grasp force time-series from all participants in all movement directions without time normalization. Each thin trace corresponds to data from one participant. Dots indicate the time of peak velocity per participant. Thick traces are the group mean traces along with one standard error.
